# A Bibliometric Analysis of Research on the Links Between Gut Microbiota and Atherosclerosis

**DOI:** 10.3389/fcvm.2022.941607

**Published:** 2022-07-12

**Authors:** Ya Wang, Dandan Li, Zijun Jia, Jiaqi Hui, Qiqi Xin, Qingbing Zhou, Weihong Cong, Fengqin Xu

**Affiliations:** ^1^Institute of Geriatric, Xiyuan Hospital, China Academy of Chinese Medical Sciences, Beijing, China; ^2^Laboratory of Cardiovascular Diseases, Xiyuan Hospital, China Academy of Chinese Medical Sciences, Beijing, China; ^3^National Clinical Research Center for Chinese Medicine Cardiology, Beijing, China

**Keywords:** gut microbiota, atherosclerosis, bibliometric analysis, Citespace, VOSviewer

## Abstract

**Background:**

Emerging evidence has linked gut microbiota (GM) and its related metabolites to atherosclerosis (AS). This study aimed to analyze the evolution of GM in AS in the past decades, and provide valuable insights in this field.

**Methods:**

Web of Science Core Collection (WoSCC) was applied to retrieve the publications related to GM in AS from their inception until 2 December 2021, and the data was analyzed in Microsoft Excel, Scimago Graphica, CiteSpace, and VOSviewer.

**Results:**

In total, 560 documents were extracted from the WoSCC databases. The publications have shown rapid growth since 2008. China and Cleveland Clin were the most prolific country and institution, respectively. The journal with the most publications is *Nutrients*, and *Nature* was the most co-cited journal. Among 3556 related authors, Hazen, Stanley L., Tang, W. H. Wilson, and Wang, Zeneng were the top 3 contributing authors in this field. Aside from “gut microbiota,” “atherosclerosis,” the terms “TMAO,” “metabolite,” “obesity,” and “phosphatidylcholine” were frequently occurred in the abstract and title of articles. Burst detection of keywords indicated that “metabolic syndrome,” “acid,” and “bile acid” were hot topics in recent years. According to the co-citation analysis of references, the research focus in this area has changed over time, and recent researches focus on choline, hypertension, butyrate, and berberine.

**Conclusion:**

Our study showed that the researches of GM in AS have been flourishing, and the content themes were constantly deepened. Human GM is critical to atherosclerotic diseases, and this hot topic is still worthy of more focus in the future.

## Introduction

Atherosclerosis (AS) is a chronic inflammatory disease characterized by accumulation of fatty and fibrous material in the intimal layer of arteries (1). AS is the primary cause of atherosclerotic cardio-cerebral diseases including coronary heart disease and stroke, the two leading causes of death in the world (2). Previous studies have indicated the links between AS and the altered profile of human gut microbiota (GM). Karlsson et al. demonstrated that symptomatic AS patients were lower in *Eubacterium* and *Roseburia*, whereas enriched in *Collinsella* (3). Jie et al. performed a metagenome-wide study on stools from patients with atherosclerotic cardiovascular diseases (ASCVD) and the healthy, and they found that *Enterobacteriaceae* and *Streptococcus* spp. increased significantly in ASCVD (4). Moreover, untargeted metabolomic analysis was performed by Koeth et al. revealing that lecithin and L-carnitine can be converted into trimethylamine oxide (TMAO) under the action of intestinal flora, which finally induces AS and accelerates the pathological process of coronary and cerebrovascular diseases (5). Short chain fatty acids (SCFAs), metabolites from indigestible dietary fibers fermented by bacteria in the colon (6), possess broad effects on AS. For example, researches have shown that butyrate inhibits AS through increasing expression of transporter ATP-binding cassette transporter A-1 (ABCA-1) and cholesterol efflux (7), and suppressing the expression of inflammatory and cell adhesion factors (8). Therefore, the GM and related metabolites may serve as potential and novel targets for preventing and treatment of AS.

Different from systematic reviews, bibliometric analysis refers to an analysis of using mathematical and statistical methods to analyze publications qualitatively and quantitatively, which is conducive to identify scientific output, research hotspots, and developing trends associated with a particular field (9–11). Although a series of bibliometric studies have been conducted to evaluate research productivity regarding GM (12–17), there has not been an assessment regarding the GM in AS. Thus, our bibliometric analysis of the literature within this field will help to address the research gaps and deepen understanding of the latest perspectives of the GM in AS.

## Materials and Methods

### Data Sources and Search Strategy

**FIGURE 1:**
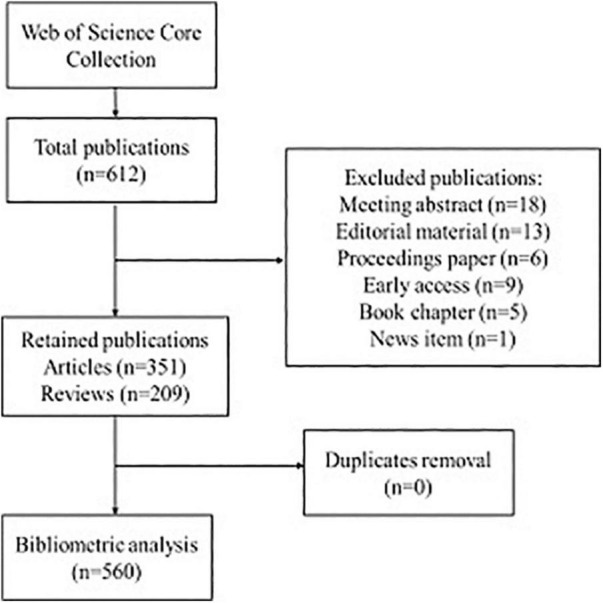
Flowchart for the publications selection included in this study.

A systematic literature search was conducted using Web of Science Core Collection (WoSCC) with no specified start date, but up to 2 December 2021. The literature was limited to the English language. Details of the search strategy were shown in Table 1. A total of 612 records were found, and the present analysis was only concerned with 2 types of documents including articles and reviews. Finally, 560 retrieval records were used for analysis, and the flow chart of literature screening was shown in Figure 1.

**TABLE 1:** Search strategy.

Rank	Search phrases	Result
#1	[(TS = atherosclerosis) OR TI = (atheroscleroses OR atherogenesis)] OR AB = (atheroscleroses OR atherogenesis)	181,328
#2	[(TS = gastrointestinal microbiome) OR TI = (gut microbiota OR gut flora OR intestinal flora OR dysbiosis OR eubiosis OR gastrointestinal microbiota OR intestinal microbiome)] OR AB = (gut microbiota OR gut flora OR intestinal flora OR dysbiosis OR eubiosis OR gastrointestinal microbiota OR intestinal microbiome)	39,387
#3	#1 AND #2	612

### Data Analysis

The data to be studied, such as yearly number of publications, and number of articles published by countries/regions, institutions, journals, and authors were downloaded from WoSCC. Additionally, the impact factor (IF) and quartile in category of journals were obtained from the 2020 Journal Citation Reports^1^ to evaluate the scientific influence of the journal. All data was imported manually into Microsoft Excel 2019 and presented in tables or charts using Excel features.

VOSviewer software can construct networks of country/region, institutions, journal, researchers, and individual publications based on citation, bibliographic coupling, co-citation, or co-authorship relations (18). It is especially useful for displaying large bibliometric maps in an easy-to-interpret way. In this study, this software was used to perform (1) countries/regions co-authorship, (2) institutions co-authorship, (3) journals citation and co-citation, (4) authors co-authorship and co-citation, and (5) keywords co-occurrence. The parameter settings of VOSviewer were as follows: type of analysis (select one at a time, such as country/region, institution, journal, author, or keywords), thresholds of items (depending on particular situations), VOSviewer thesaurus file (merge different variants of keywords).

Citespace can visually map highly cited and pivotal documents, areas of specialization within a knowledge domain, and emergence of research topics (19). In this study this software was used to (1) detect a citation-burst analysis of references and keywords, (2) generate the references visualization map from the cluster and time zone view, (3) generate the co-cited authors collaboration network visualization map. The betweenness centrality feature is used to measure the importance of a node in the network and a node with higher centrality value is recognized a core point or pivotal turning point in a field (10, 11). For Citespace, the parameter settings were as follows: time span (1995–2021), years per slice (2), node type (choose one at a time, such as keyword, reference, or cited author), term source (title, abstract, author keywords, keywords plus), selection criteria (threshold: c, cc, and ccv depending on particular situations), pruning (pathfinder, pruning sliced networks), and visualization (default parameters). More pertinent conclusions could be obtained according to particular time slices and threshold settings.

Apart from the above methods, the Scimago Graphica tool was used to perform collaboration network between countries/regions and institutions.

## Results

### Analysis of Publication Trends

**FIGURE 2:**
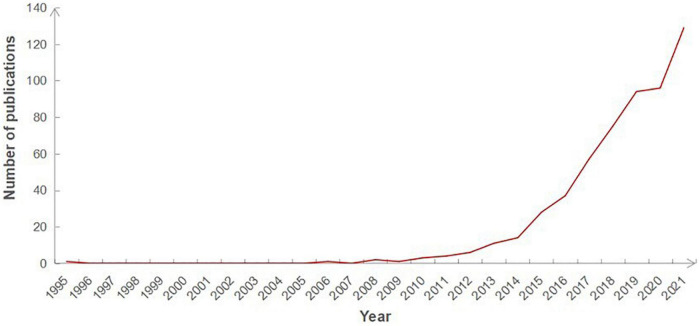
The number of publications annually related to GM in AS.

Annual publications can be used to reflect the development profile of a certain field. Figure 2 shows an overall uptrend in this field. The total research output was very low before 2008, but the annual output subsequently showed rapid upward trends. Although there was a short platform period during 2019–2020, since then, the number of publications in this field has grown rapidly. It can be seen that increasingly scholars have begun to pay much attention to the potential of intestinal flora in the treatment of diseases associated with AS.

### Analysis of International and Institutions Collaborations

A total of 560 documents were published by 945 institutions in 59 countries/regions, and the top 10 most productive countries/regions or institutions are shown in Tables 2, 3. Our study indicated that China (190, 33.93%) was the most productive country, followed by United States (107, 19.11%), Italy (26, 4.64%), Japan (26, 4.64%), and Canada (20, 3.57%). The most active affiliate was Cleveland Clin (28, 5.0%), followed by Univ Copenhagen (15, 2.68%), Univ Calif Los Angeles (14, 2.5%), Univ Gothenburg (11, 1.96), and Oslo Univ Hosp (10, 1.79%). Total link strength (TLS) is an important indicator to quantitatively assess the correlation strength between countries/regions and institutions (11). By assessing the TLS of countries/regions and organizations, we identified United States and Cleveland Clin as the most influential country and institution in this field.

**TABLE 2:** Top 10 countries/regions with the largest number of outputs or the largest cooperation intensity.

Rank	Country/region	Counts (%)	Co-authorship country/region	Total link strength
1	China	190 (33.93)	United States	78
2	United States	107 (19.11)	China	40
3	Italy	26 (4.64)	Italy	36
4	Japan	26 (4.64)	United Kingdom	36
5	Canada	20 (3.57)	Germany	29
6	Germany	19 (3.39)	Sweden	23
7	Spain	16 (2.86)	Switzerland	23
8	Iran	14 (2.80)	France	22
9	Sweden	13 (2.32)	Spain	20
10	Netherlands	12 (2.14)	Denmark	18

**TABLE 3:** Top 10 active institutions with the largest number of articles or the largest cooperation intensity.

Rank	Institution	Counts (%)	Co-authorship institution	Total link strength
1	Cleveland Clin	28 (5.00)	Cleveland Clin	31
2	Univ Copenhagen	15 (2.68)	Univ Calif Los Angeles	24
3	Univ Calif Los Angeles	14 (2.50)	Oslo Univ Hosp	20
4	Univ Gothenburg	11 (1.96)	Univ Oslo	18
5	Oslo Univ Hosp	10 (1.79)	Cleveland State Univ	17
6	Southern Med Univ	10 (1.79)	Univ Copenhagen	15
7	Sun Yat-sen Univ	10 (1.79)	Univ N Carolina	15
8	Univ Oslo	10 (1.79)	Univ Bergen	14
9	Cleveland State Univ	9 (1.61)	Univ So Calif	13
10	Kobe Univ	9 (1.61)	Univ Wisconsin	10

**FIGURE 3:**
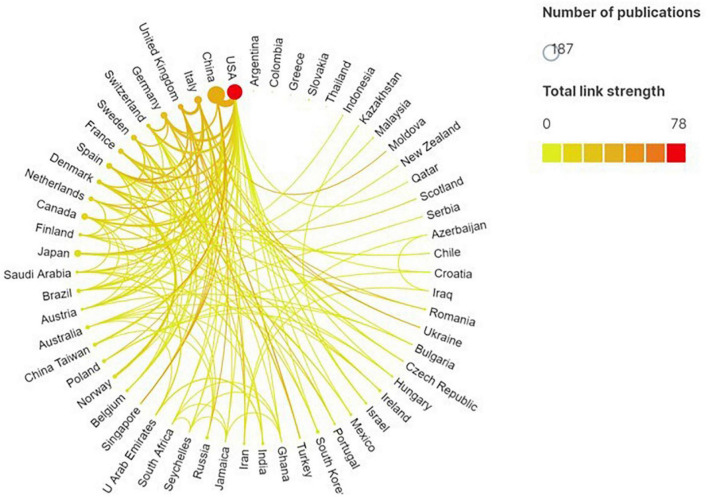
Country/region collaboration map generated by Scimago Graphica software. Each node represents a country/region, and the node size is proportional to the number of the publications. Line thickness between nodes indicates link strength of a collaboration relationship (weighted by a quantitative evaluation indicator of total link strength).

**FIGURE 4:**
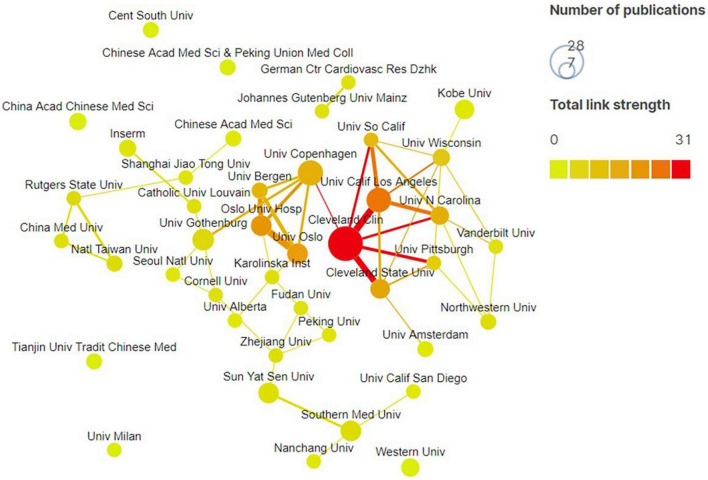
Institution collaboration map generated by Scimago Graphica software. Each node represents an institution, and the node size is proportional to the number of the publications. Line thickness between nodes indicates link strength of a collaboration relationship (weighted by a quantitative evaluation indicator of total link strength).

The cooperation maps by the country/region and institution were shown in Figures 3, 4. The size of each node represents the number of documents. The link line width were proportional to the tightness of the cooperation between the nodes. The wider the line, the closer the cooperation. We can see that the United States works with many countries, and most closely with China. Moreover, many research institutions also cooperated actively, especially Cleveland Clin.

### Analysis of Active Journals and Co-cited Academic Journals

The documents included were published in 283 journals. Total citation (TC) represents the total number of citations for a journal. As shown in Table 4, most of the top 10 relevant journals had IFs above 4: *Nutrients* (IF = 5.719_2020_, *N* = 21, TC = 458), *Scientific Reports* (IF = 4.38_2020_, *N* = 15, TC = 230), *Journal of Agricultural and Food Chemistry* (IF = 5.279_2020_, *N* = 12, TC = 139), *Frontiers in Pharmacology* (IF = 5.811_2020_, N = 11, TC = 240), *International Journal of Molecular Sciences* (IF = 5.924_2020_, *N* = 11, TC = 308). And we found that there was an insignificant positive correlation between the total number of journals published and the TCs.

**TABLE 4:** Top 10 journals and co-cited journals related to GM in AS.

Rank	Journal	Count (%)	Total citations (TC)	IF (2020)	JCR	Co-cited journal	Total citations (TC)	IF (2020)	JCR
1	Nutrients	21 (3.75)	458	5.719	Q1	Nature	1662	49.962	Q1
2	Scientific Reports	15 (2.68)	230	4.38	Q1	Plos One	1036	3.24	Q2
3	Journal of Agricultural and Food Chemistry	12 (2.14)	139	5.279	Q1	Proceedings of the National Academy of Sciences of the United States of America	976	11.205	Q1
4	Frontiers in Pharmacology	11 (1.96)	240	5.811	Q1	Circulation	753	29.69	Q1
5	International Journal of Molecular Sciences	11 (1.96)	308	5.924	Q1	Cell	676	41.584	Q1
6	Plos One	11 (1.96)	405	3.24	Q2	Nature Medicine	635	53.44	Q1
7	Food and Function	10 (1.79)	124	7.514	Q1	New England Journal of Medicine	612	91.254	Q1
8	Molecular Nutrition and Food Research	9 (1.61)	314	5.82	Q1	Cell Metabolism	589	27.287	Q1
9	Nutrition Metabolism and Cardiovascular Diseases	9 (1.61)	132	4.222	Q2	Atherosclerosis	580	5.162	Q2
10	Atherosclerosis	8 (1.43)	439	5.162	Q2	Science	575	47.728	Q1

**FIGURE 5:**
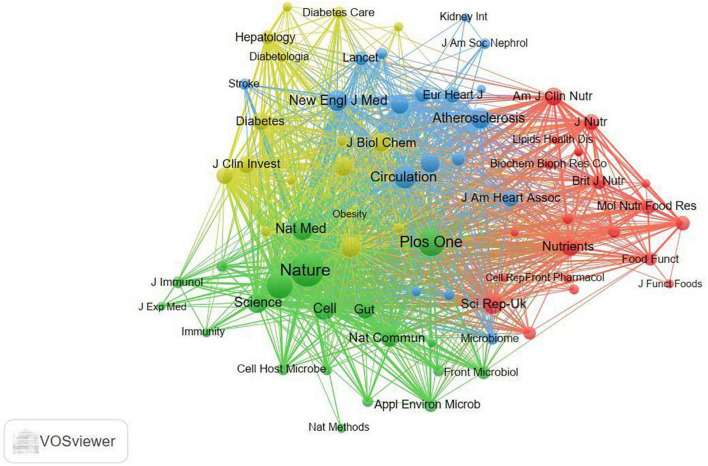
Visualization map of the journal co-citation analysis generated by VOSviewer software. Each node represents a journal, and the node size is proportional to the number of the co-citations.

Journal co-citation analysis, first introduced by McCain (20), focuses primarily on the relationship between journals, which reflects whether the journal acts as a bridge in a particular research field (21). With the development of scientometric research such as scientific knowledge map and visualization technology, journal co-citation analysis has increasingly become a hot spot. From Table 4, it can be seen that there were 2 academic journals with more than 1000 citations, 1 is *Nature* (IF = 49.962_2020_) and the other is *Plos One* (IF = 3.24_2020_). Figure 5 demonstrates a strong tendency toward co-citation relationships between the journal *Nature* and other journals.

### Analysis of Active Authors and Co-cited Authors

**FIGURE 6:**
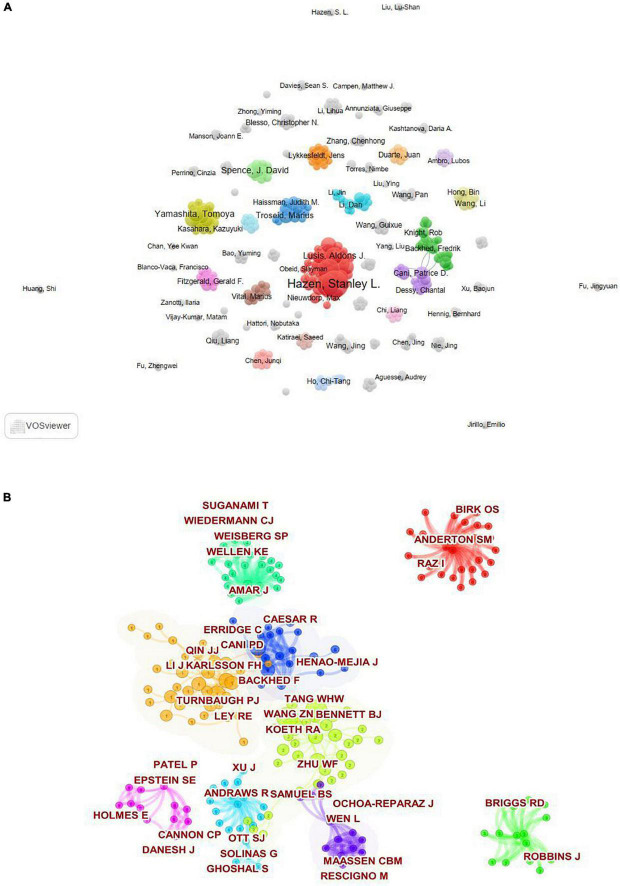
**(A)** Inter-author collaborative map generated by VOSviewer software. **(B)** The co-cited authorship clustering analysis generated by Citespace software.

A total of 3556 authors were included in this field. The top 10 authors who contributed and were cited most are shown in Table 5, respectively. The 3 most prolific authors from the top 10 authors are Hazen, Stanley L. (25, 4.46%), Tang, W. H. Wilson (17, 3.04%), and Wang, Zeneng (17, 3.04%). As showed in the author co-occurrence visualization network map (Figure 6A), there has been many academic teams in this field. Co-cited authors refers to that two or more authors are simultaneously cited in one or more subsequent papers, which is called co-cited relationship between these two or more authors (22). Author co-citation analysis can discover highly influential scholars in a discipline area. The top 10 co-cited scholars were listed in Table 5. In terms of the number of citations, Wang ZN ranked first, with 286 citations, followed by Tang WHW (citations = 278), Koeth RA (citations = 247), Turnbaugh PJ (citations = 143), and Cani PD (citations = 137). Top three of them come from Cleveland Clin. The cluster-based analysis of co-cited authors carried out by Citespace was showed in Figure 6B. The size of each node represents the number of publications. The link line width were proportional to the tightness of the cooperation between the nodes. The wider the line, the closer the cooperation. In addition, nodes belonging to a cluster have the same color, which means these authors have the same research direction.

**TABLE 5:** Top 10 authors and co-cited authors related to GM in AS.

Rank	Author	Count (%)	Co-cited author	Citation	Centrality
1	Hazen, Stanley L.	25 (4.46)	Wang ZN	286	0.22
2	Tang, W. H. Wilson	17 (3.04)	Tang WHW	278	0.02
3	Wang, Zeneng	17 (3.04)	Koeth RA	247	0.07
4	Lusis, Aldons J.	10 (1.79)	Turnbaugh PJ	143	0.11
5	Didonato, Joseph A.	9 (1.61)	Cani PD	137	0.27
6	Spence, J. David	9 (1.61)	Karlsson FH	130	0.09
7	Wu, Yuping	9 (1.61)	Zhu WF	121	0.09
8	Yamashita, Tomoya	9 (1.61)	Bennett BJ	111	0.09
9	Bennett, Brian J.	8 (1.43)	Qin JJ	109	0.03
10	Hirata, Ken-Ichi	8 (1.43)	Ley RE	108	0.11

### Keywords Co-occurrence Analysis and Burst Analysis

**FIGURE 7:**
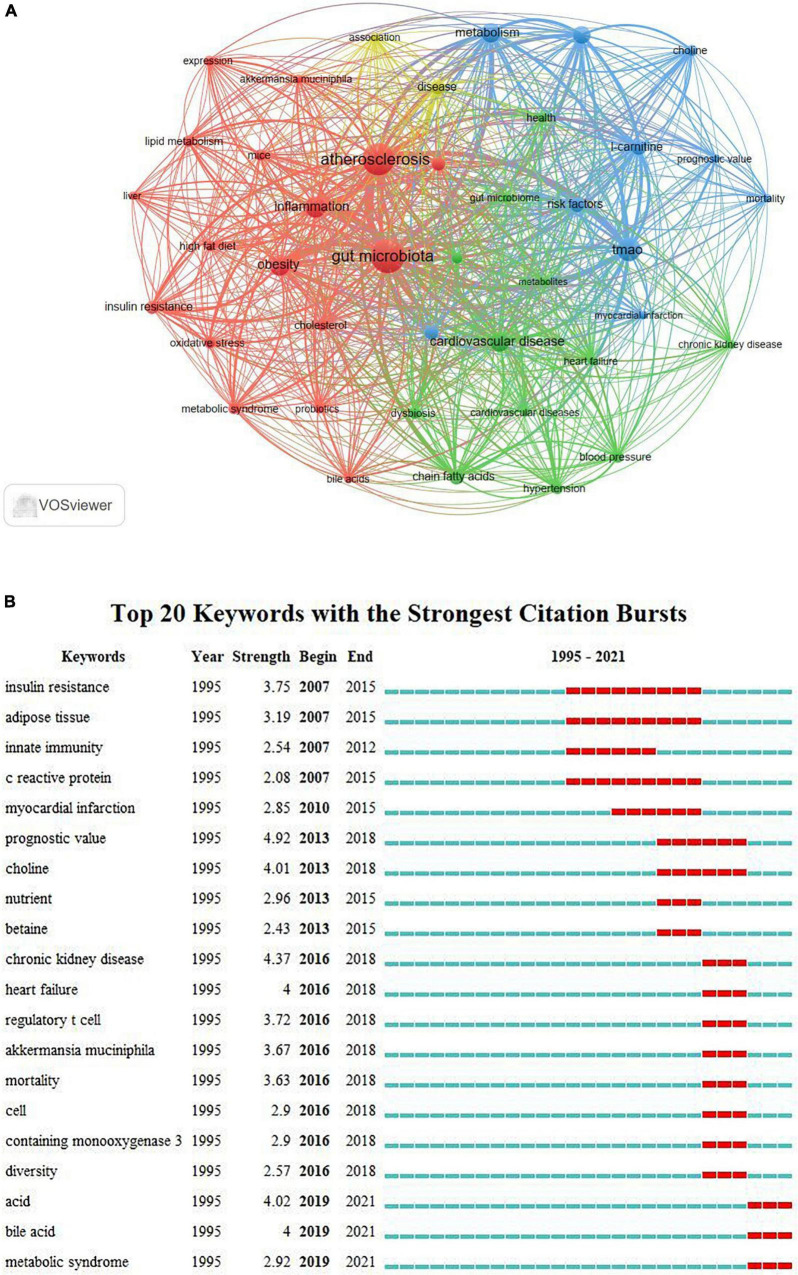
Top 20 keywords with the strongest citation bursts related to GM in AS.

Keywords reflect the topic of an article, which can be identified to analyze research hotspots and frontiers in a field. The keywords co-occurrence visualization map was constructed in VOSviewer software, and 41 high-frequency keywords (more than 20 times) were included (Figure 7A). We can see from the Figure 7A, all keywords could be classified into three clusters: cluster 1 (mechanism and therapy), cluster 2 [cardiovascular disease (CVD)], and cluster 3 (choline and coronary artery disease). Table 6 presents the top 30 highest frequency keywords related to GM in AS. Aside from “gut microbiota” and “atherosclerosis,” the terms “TMAO,” “metabolite,” “obesity,” and “phosphatidylcholine” frequently occurred in the abstract and title of articles. Betweenness centrality is an indicator to measure the importance of nodes in the network, nodes whose centrality over 0.1 are called critical nodes. In this study, “TMAO,” “obesity,” and “health” are the three most important keywords besides “gut microbiota” and “atherosclerosis,” whose centrality are greater than 0.2.

**TABLE 6:** Top 30 keywords related to GM in AS.

Rank	Keyword	Count	Centrality	Rank	Keyword	Count	Centrality
1	Gut microbiota	235	0.35	16	Cholesterol	25	0.06
2	Atherosclerosis	189	0.37	17	Bile acid	25	0.03
3	TMAO	141	0.28	18	High fat diet	23	0.19
4	Metabolite	118	0.09	19	Oxidative stress	21	0.12
5	Obesity	103	0.29	20	Mortality	21	0.02
6	Phosphatidylcholine	101	0.06	21	Acid	20	0.03
7	Inflammation	95	0.01	22	*Akkermansia muciniphila*	19	0.02
8	Cardiovascular disease	82	0.16	23	Dysfunction	17	0.09
9	L-Carnitine	74	0.12	24	Choline	17	0.07
10	Chain fatty acid	69	0.10	25	Heart failure	15	0.10
11	Diet	52	0.09	26	Chronic kidney disease	14	0.03
12	Insulin resistance	42	0.14	27	Prognostic value	14	0.10
13	Coronary artery disease	40	0.15	28	Farnesoid X receptor	14	0.03
14	Blood pressure	37	0.10	29	Metabolic syndrome	13	0.00
15	Health	34	0.25	30	Endotoxemia induced inflammation	12	0.08

Keywords burst detection can find the research hotspots increasing abruptly in a specific field. The strength of the burst and the duration of the burst state are the two attributes of the citation burst (23). Based on keyword burst analysis, the top 20 keywords with the strongest citation burst from 1995 to 2021 as shown in Figure 7B. In addition, “metabolic syndrome,” “acid,” and “bile acid” were hot topics in recent years among these keywords with a burst strength of 2.92, 4.02, and 4, respectively.

### Co-citation Analysis of References

Co-citation references are those simultaneously cited by one or more papers, and can be viewed as the knowledge bases in a certain field. When they were frequently cited at the same time, indicating these studies were highly correlated and had similar research topic. Table 7 presents the top 10 most frequently cited references, and the paper authored by Koeth et al. (5) is the most highly cited. The article was the first to demonstrate that dietary L-carnitine from red meat can be metabolized to a pro-atherosclerotic harmful metabolite, TMAO, under the action of intestinal flora. In addition, it suggested one pro-atherosclerotic mechanism of TMAO was diminishing reverse cholesterol transport (RCT). Additionally, “*Trimethylamine-N-oxide, a metabolite associated with atherosclerosis, exhibits complex genetic and dietary regulation*” and “*Symptomatic atherosclerosis is associated with an altered gut metagenome*” were two key references in this field with a centrality of 0.1, separately. The former (24) demonstrated that flavin mono-oxygenase 3 (FMO3) had a highly specific activity of oxidizing trimethylamine (TMA) to TMAO, and levels of TMAO and FMO3 expression were regulated by the nuclear receptor—farnesoid X receptor (FXR). The study provided evidence for targeting regulation of TMAO levels within the circulation. The later study (3) identified that patients with symptomatic atherosclerosis harbor characteristic changes in the intestinal flora, with increased *Collinsella* and decreased *Roseburia* and *Eubacterium*.

**TABLE 7:** Top 10 most co-cited references related to GM in AS.

Rank	Title	Citation	Centrality	Year	Source	References
1	Intestinal microbiota metabolism of L-carnitine, a nutrient in red meat, promotes atherosclerosis.	235	0.05	2013	Nature Medicine	(5)
2	Intestinal microbial metabolism of phosphatidylcholine and cardiovascular risk.	199	0.01	2013	New England Journal of Medicine	(37)
3	Gut flora metabolism of phosphatidylcholine promotes cardiovascular disease.	161	0.02	2011	Nature	(36)
4	Gut microbial metabolite TMAO enhances platelet hyperreactivity and thrombosis risk.	123	0.05	2016	Cell	(81)
5	Non-lethal inhibition of gut microbial trimethylamine production for the treatment of atherosclerosis.	119	0.02	2015	Cell	(82)
6	Trimethylamine-N-oxide, a metabolite associated with atherosclerosis, exhibits complex genetic and dietary regulation.	109	0.1	2013	Cell Metabolism	(83)
7	Symptomatic atherosclerosis is associated with an altered gut metagenome.	94	0.1	2012	Nature Communications	(3)
8	The gut microbiome in atherosclerotic cardiovascular disease.	80	0.04	2017	Nature Communications	(4)
9	Transmission of atherosclerosis susceptibility with gut microbial transplantation.	77	0.05	2015	Journal of Biological Chemistry	(84)
10	Resveratrol attenuates trimethylamine-N-oxide (TMAO)-induced atherosclerosis by regulating TMAO synthesis and bile acid metabolism *via* remodeling of the gut microbiota.	75	0.01	2016	mBio	(25)

**FIGURE 8:**
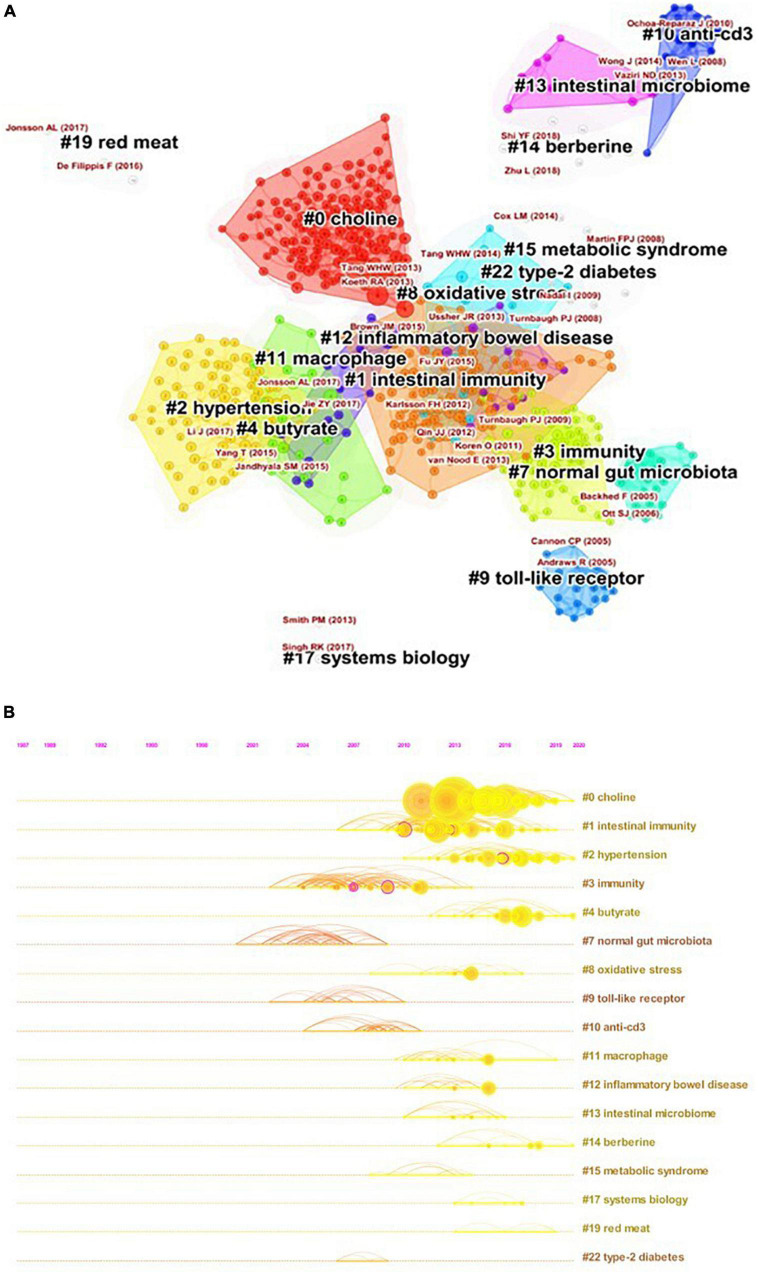
The analysis of co-citation references in the field of GM in AS. **(A)** The network map of co-citation clusters. Seventeen clusters with different research topics were formed, reflecting in different colors on the map. **(B)** Timeline visualization of co-citation clusters. Each horizontal row represented a cluster, and each node on the line denoted a co-citation reference. The co-citation relationship between the two references is represented as line connecting two nodes, and the size of the node meant the number of the co-cited times.

On the basis of keywords extracted from the references, 17 different sizes and colors clusters were formed (Figure 8A), meaning that 17 different research topics have been formed in this field. Figure 8B presents evolution of these clusters on a timeline, revealing that clusters #0, #2, #4, and #14 were identified as the most recent regions. As shown in Figure 8B, all cited references were from 2004 to 2020, indicating the rapid development in this area.

**FIGURE 9:**
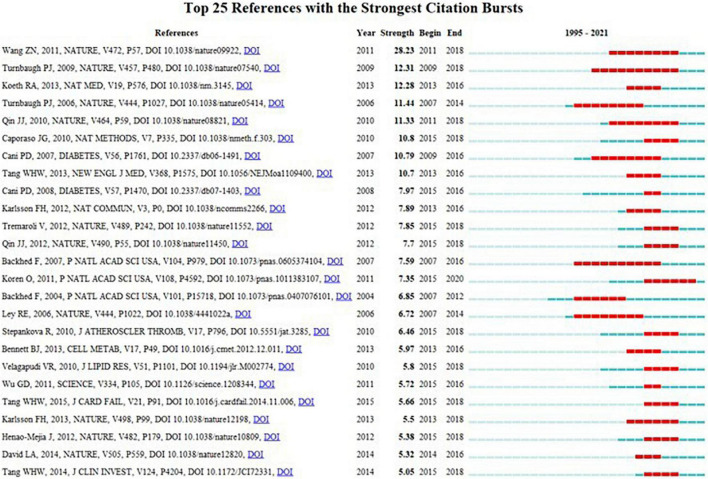
Top 25 references with the strongest citation bursts related to GM in AS.

Burst detection of references reflects the change of research focus in a specific area over time. Among the top 25 references with the strongest citation bursts (Figure 9), the article authored by Koren et al. (25) with a burst strength of 7.35 was frequently cited in recent years. Koren et al. investigated the bacterial diversity of atherosclerotic plaque, oral, and gut samples of 15 patients with AS. They found that *Chryseomonas* was in all atherosclerotic plaque samples, moreover, several bacterials were common in the atherosclerotic plaque, oral, or gut samples from the same individual. They also revealed that both oral and gut microbiota were associated with disease biomarkers such as serum cholesterol.

## Discussion

Cardiovascular disease is the leading cause of global mortality and disability, and is the major contributor to rising health care costs worldwide (26). As the main underlying etiology for CVD, it is very important to delay or reverse the progression of AS. Evidence for the importance of GM in human health is growing (27), and studies also have shown dysbiosis of GM linked to multiple atherosclerotic diseases (28, 29). Given the significant role in host health, GM has gained increasing attention. Therefore, it is necessary to make a bibliometric analysis focusing on GM in the field of AS.

### General Information

According to the current analysis, there was particular little literature published before 2008, suggesting GM has not been heavily investigated in research on AS. However, from 2008 to present, the increasing number of annual publications indicates that this research area is a hotspot, and continues to receive attention. In consistent with previous studies (23, 30, 31), the annual number of articles showed rapid growth not only in the whole field of human gut microbiota research, but also in the field of GM related to other CVDs. It indicates that the popularity of gut microbiome field will continue to increase.

Visual analysis of the distribution of country/region and institution shows that many countries and regions have studied the human gastrointestinal microbiome. China has contributed the most to publications in this field, which may be attached to the key research and development project of biomacromolecules and microbiome issued by the Ministry of Science and Technology of China (32). Although China was not the first country to study human intestinal flora, its exploration in this field is deepening. This is especially true of scholars from Southern Medical University and Sun Yat-sen University, which have the largest number of publications. The second most published country is United States, which may be attributed to the two important project, the Human Microbiome Project (HMP) and the Gut Microbiota Brain AXIS program (33, 34). However, in terms of the entire GM field, the United States was the most productive country (12, 30). Additionally, two bibliometric analyses for GM in heart failure (23) and hypertension (HTN) (30) also demonstrated that the United States with the most publications. Many factors may contribute to this disparity, funding support may be one of them. Notably, although the United States published less than China, it dominates this field. As we can see from the Table 2, the United States had the largest TLS, which indicated that articles published in the United States may be more influential in this field. Similar to our study, a scientometric analysis related to GM is heart failure identified the United States was actually dominated in the field using centrality value (23).

There are nearly 1000 institutions around the world have been studying the GM in AS. We can see from Table 3, Cleveland Clin was the most productive and most influential institution which located in the United States. This may be attributed to contributions made by the scholars, such as Hazen, Stanley L., Wang ZN, and Tang, W. H. Wilson, of this institution. Together, these may have promoted the United States dominance in this field. Meanwhile, other bibliometric analysis also demonstrated that Cleveland Clin was the most productive institution (23). These results indicated that establishing top-notch research institutions is conducive to enhancing international academic status of a country. Notably, as in Figure 4, although part of these institutions have cooperated closely, some have not. Thus, it is strongly suggested that countries and institutions with similar research topic should strengthen the breadth and depth of cooperation, and jointly promote the prosperity and development of this field.

The top 10 journals may be preferentially selected when researchers peruse and publish related articles, because they have published many articles in this field. As showed in Table 4, most of the top 10 journals belong to the JCR Q1 zone, with IFs between 3.0 and 8.0, which indicates a relatively high quality of literature in this field. However, a study by Yuan et al. (31) showed the top 10 most published journals related to human gastrointestinal microbiome during 2010–2021 including 2 high IFs (more than 10), *Microbiome* and *Gut*. It implies that publishing articles related to GM in AS in high IFs journals may be a challenge. According to our analysis, those with high co-citations articles were distributed in multiple high-profile journals, such as *New England Journal of Medicine*, *Cell*, *Nature*, *Science*, and *Circulation*. It suggested that GM in AS have attracted considerable attention from top scholars because of its potential impact on host health. Additionally, Mu et al. (23) carried out a bibliometric analysis to explore the links between GM and heart failure, and they also demonstrated that *New England Journal of Medicin*e, *Cell*, *Nature*, *Science*, *Circulation*, *Proceedings of the National Academy of Sciences of the United States of America*, and *Plos One* were highly cited journals. Thus, more attention should focused on these journals to obtain new research progress or discovery.

It is obvious that among numerous scholars in this field, Hazen, Stanley L., Tang, W. H. Wilson, and Wang, Zeneng have contributed the most, which is related to the large amount of literature they have published. Different from our result, the study by Mu et al. (23) showed that Hazen, Stanley L., and Tang, W. H. Wilson only published less than 10 articles in the field of GM and heart failure. From the visual map of author cooperation network, we can see, although a large number academic teams have been formed in this field, there is little cooperation among different teams. This situation is not conducive to the development of scientific research. It is strongly recommended that all academic teams strengthen scholarly communication, deeply explore the connection between GM and host cardiometabolic health, and jointly promote the rapid development of this field.

It is noteworthy that Wang Zeneng, the most co-cited author in this field, with the centrality of 0.22, has a strong academic influence and makes significant contributions to this field. Additionally, a bibliometric analysis to trace the global trends in metabolomics in coronary artery disease performed by Yu et al. (35) also showed that Wang Zeneng and Tang WHW were highly co-cited authors. This may be attributed to the fact that microbiomics is often used in conjunction with metabolomics. Wang et al. proved the association between higher plasma levels of choline, TMAO, and betaine and the risk of AS in humans. In addition, they also demonstrated the pro-atherosclerosis mechanism of these substances through mice experiment, establishing a solid foundation for the relationship between intestinal flora and AS (36).

### Hotspots and Frontiers

The most valuable information that bibliometric analysis can provide is the knowledge base and research frontier in a particular field, which can be reflected by literature co-citation analysis and burst detection, respectively. Analysis of timeline view (Figure 8B) showed that the research focus in this area has changed over time, with recent focus including choline (cluster #0), hypertension (cluster #2), butyrate (cluster #4), and berberine (cluster #14).

Among numerous research efforts on these topics, the work of Tang and Koeth has had a profound academic impact on this field since their publication in *The New England Journal of Medicine* in 2013 or *Nature Medicine* in 2013. The two most cited articles in cluster #0. In 2011, Wang et al. demonstrated there was a strong causal relationship between TMAO and AS (36). The team then found that increased TMAO levels are related to an increased risk of incident major adverse cardiovascular events (37). Koeth et al. reported that intestinal microbiota could metabolize L-carnitine into TMA, which is oxidized in the liver into TMAO, and mice treated with TMAO or either carnitine or choline reduced RCT *in vivo* (5). From then on, TMAO as an independent risk factor for CVD, has courted considerable research interest. Skye et al. demonstrated that *CutC* is a lyase that converts choline to TMA, the precursor of TMAO, which may be a potential molecular target for the treatment of atherothrombotic heart disease (38). In 2019, Koeth et al. suggested that sequential microbial reaction of L-carnitine → γ-butyrobetaine → TMA, was a major pathway for orally ingested L-carnitine conversion into TMA and thus, TMAO (39). This study provided important insights for efforts aimed at development of therapeutic interventions for preventing or treating AS. However, several recent studies have reported results different from previous. Lindskog Jonsson et al. reported that choline supplementation increased plasma TMAO concentrations in male mice, but no change in AS was observed (40). Consistent with the finding, Aldana-Hernández et al. also found that high intakes of dietary choline or TMAO supplementation did not affect the development of AS in Ldlr^–/–^ or ApoE^–/–^ male mice (41). Thus, substantial work is required to understand the physiological consequences of TMAO in mammals.

Hypertension is the global public health concern and a major risk factor for AS, which has been the focus of scholars. In our study, we found that HTN has been one of the research hotspots in recent years in the field of GM in AS, and most studies in this cluster distributed in the United States. Interestingly, a recent bibliometric analysis explored the worldwide research trends in HTN, and it showed that the United States also has been the most contributor in the field of HTN (42). Yang et al. (the most cited article in cluster #2) firstly proved that HTN is related to altered GM in two different rat models of HTN and a small cohort of HTN patients (43). Their work implicated the role of gut flora in the pathophysiology of HTN, providing a basis for further research. A cohort study by Li et al. demonstrated that pre-hypertensive and hypertensive populations had significantly different GM profile compared with healthy control group, and their work of fecal bacteria transplantation firstly established the causal link between gut dysbiosis and HTN (44). Karbach et al. reported that GM could promote Ang II-related HTN, partly due to immune activation (45). In addition to GM, TMAO also promote Ang II-induced HTN by stimulating the PERK/ROS/CaMKII/PLCβ3 axis (46). Possible mechanisms by which intestinal flora and their metabolites promote HTN are complex, and much research is needed in the future.

Short chain fatty acids are products of dietary fiber fermented by intestinal flora, mainly including acetate, propionate, and butyrate. As GM derived metabolites, SCFAs has also gradually attracted the interest of many scholars. A review by Koh et al. discussed how SCFAs are synthesized, are distributed, and contribute to host physiology, which gave us an initial understanding of the function of SCFAs (47). Among SCFAs, butyrate has received most attention for its beneficial effect on health. In 2017, the CANTOS trial carried out by Ridker et al. confirmed that anti-inflammatory treatment is beneficial to improve atherosclerotic disease and lead to lower recurrence rate of cardiovascular events (48). The research provided direct evidence that inhibition of vascular inflammation reduces cardiovascular events. Chang et al. showed that butyrate has immunomodulatory effect on intestinal macrophages by inhibiting histone deacetylases, and reduces the release of pro-inflammatory mediators (49). The pre-clinical trial of Aguilar et al. found that butyrate can delay the progression of AS by reducing CD36 in macrophages and endothelial cells, decreasing pro-inflammatory cytokines, and lowering the activation of NFκB (50). Research showed that butyrate enters colonic epithelial cells through solute carrier (SLC) transporters to upregulate tight junctions and maintain gut barrier function, thereby preventing endotoxin molecules such as lipopolysaccharide (LPS) from entering the circulation and preventing the occurrence of systemic inflammation (51). Moreover, butyrate also attenuates AS by regulating lipid metabolism through inhibiting cholesterol efflux in macrophages (7). Moderately increasing butyrate in the host possibly is a promising therapeutic strategy for protecting against AS.

Berberine, a natural isoquinoline alkaloid, extracted from Chinese herbs such as *Coptis chinensis* (52). Its anti-atherosclerosis mechanism has been well illuminated in several studies (53–56). Additionally, basic studies have showed that the mechanism was also closely related to GM. *Akkermansia muciniphila* is a gram-negative anaerobic bacteria, which scarcity can lead to diverse clinical disorders, including AS (57). Li et al. confirmed that western diet consumption reduces the abundance of *A. muciniphila* in the gut of ApoE^–/–^ mice, increases gut permeability with subsequent translocation of bacterial derived LPS, which leads to elevated LPS in serum levels. Finally, systemic inflammatory responses induced by LPS contribute to AS (58). Zhu et al. (the most cited article in cluster #14) revealed that berberine can anti-atherosclerosis by modulating the intestinal flora disturbance induced by high-fat diet, especially the abundance of *Akkermansia* (59). Shi et al. found that berberine not only restores GM profiles, but also reduces hepatic FMO3 expression and serum TMAO levels (60). Wu et al. also demonstrated that berberine could modulate the composition of the microbiota related to glycolipid metabolism and SCFAs production (61). Additionally, other bibliometric reviews published to trace the global trends of berberine (62, 63), found that “inflammation,” as well as “metabolic syndrome” are two research hotspots and frontiers of berberine. Both could be improved by berberine through regulating intestinal flora. Ever-increasing evidence suggests that berberine plays a crucial role in modulating gut dysbiosis and its metabolites, and these studies provided a solid evidence for rational treatment of AS by berberine.

Keywords with citation bursts can be used to roughly reflect the research frontier of a certain knowledge domain (19). According to our analysis, there has been considerable interest in the potential role of bile acids (BAs) in the development of AS in recent years, which has a relatively high burst strength. BAs are a family of endogenous metabolites synthesized from cholesterol in liver and then modified by enzymes produced microbiota in gut (64), which serve as versatile endocrine signaling molecules that activate multiple nuclear and membrane receptor signaling pathways (65). Among these receptors, the FXR and the Takeda G protein-coupled receptor 5 (TGR5), have been extensively studied.

As is well known, abnormal lipid metabolism plays a key role in AS. Zhang et al. demonstrated that activation of FXR decreases serum high density lipoprotein (HDL) level by inducing scavenger receptor group B type 1 (SR-BI) expression as well as increases macrophage RCT (66). In their follow-up study, they found that activation of FXR inhibits intestinal cholesterol absorption by regulating the bile acid pools, resulting in increased RCT (67). Phospholipid transfer protein (PLTP), a liver-derived protein, is important for lipoprotein metabolism (68), and study suggested that FXR regulates HDL metabolism *via* increasing expression of PLTP gene (69). Furthermore, FXR also influences HDL levels by inhibiting human apolipoprotein A-I (APOA1) gene expression (70). A FXR/ApoE double-null mice model established by Guo et al. indicated that FXR affects the development of AS by altering the uptake of oxidized low-density lipoprotein by macrophages as well as modulating the immune response (71). Apolipoprotein C-II (apoC-II), as a cofactor for the activation of lipoprotein lipase (LPL), is a critical regulator of lipoprotein metabolism (72). FXR also was shown to enhance the expression of apoC-II and reduce the plasma triglyceride (TG) levels (73).

Takeda G protein-coupled receptor 5, a member of G-protein coupled receptor (GPCR) family, which was discovered in 2002 (74). It has been identified as an important component in bile acid-regulated energy homeostasis, glucose homeostasis, and lipid metabolism (75). Obesity is a major risk factor for CVD, and contributes to increase of cardiovascular mortality *via* increased atherosclerotic burden (76). Current evidence shows that BAs increase energy expenditure in brown adipose tissue of mice, which dependent on activation of the TGR5 (77). Disturbance of glucose metabolism is another risk factor for AS. One study has shown that activation of TGR5 by a semi-synthetic cholic acid derivative improves glucose homeostasis. This mechanism is associated with TGR5 stimulating glucagon-like peptide-1 (GLP-1) release from intestinal endocrine L cells by enhancing mitochondrial oxidative phosphorylation and calcium influx (78). Another study *in vivo* showed that activation of TRG5 achieves a reduction in many lipid parameters, such as plasma TG and low-density lipoprotein cholesterol (LDL) (79). Moreover, the experimental evidence available demonstrated that the protective effects of TGR5 activation on AS may be attributed to inhibition of macrophage NFκB signaling, as well as a reduction of macrophage foam cell formation (80). Given the beneficial effects of FXR or TGR5 activation on AS, they might be potentially promising targets for AS.

There are some limitations in our study. First, in the study only literature that in WoSCC database were retrieved, and it is possible that related studies to be missed, which may influence the results of this study. Second, we only included articles and reviews in the English language, which may lead to bias. Third, data downloaded was not full text, some important information may be omitted.

## Conclusion

To our knowledge, this is the first study to use bibliometric analysis to explore the development of GM in AS. A vast amount articles in this field has increased in recent years, meaning the role of GM in AS has received considerable scholarly attention. However, the scientific cooperation network indicated that collaboration between different institutions and academic teams has not been sufficient. With the deepening of the research in this field, there have been several different research topics gradually formed. According to our analysis, bile acids have been the research frontier in this field recently, which may be a potential therapeutic target for AS. All in all, the study of GM is an ongoing research hotspot and contributes to preventing or treating ASCVD.

## Data Availability Statement

The original contributions presented in the study are included in the article/supplementary material, further inquiries can be directed to the corresponding authors.

## Author Contributions

YW and QZ conceived the study. DL and ZJ collected the data. QZ and JH re-examined the data. YW, DL and QX analyzed the data. YW wrote the manuscript. QZ, WC, and FX reviewed and revised the manuscript. All authors contributed to the article and approved the submitted version.

## Conflict of Interest

The authors declare that the research was conducted in the absence of any commercial or financial relationships that could be construed as a potential conflict of interest.

## Publisher’s Note

All claims expressed in this article are solely those of the authors and do not necessarily represent those of their affiliated organizations, or those of the publisher, the editors and the reviewers. Any product that may be evaluated in this article, or claim that may be made by its manufacturer, is not guaranteed or endorsed by the publisher.

## Footnotes

1
http://clarivate.com/products/web-of-science
